# Pulmonary Endogenous Fluorescence Allows the Distinction of Primary Lung Cancer from the Perilesional Lung Parenchyma

**DOI:** 10.1371/journal.pone.0134559

**Published:** 2015-08-05

**Authors:** Lucile Gust, Alexis Toullec, Charlotte Benoit, René Farcy, Stéphane Garcia, Veronique Secq, Jean-Yves Gaubert, Delphine Trousse, Bastien Orsini, Christophe Doddoli, Helene Moniz-Koum, Pascal Alexandre Thomas, Xavier Benoit D’journo

**Affiliations:** 1 Department of Thoracic Surgery and Diseases of the Oesophagus, North Hospital, Aix-Marseille University, Marseille, France; 2 Nodea Medical, Villejuif, France; 3 LAC, Paris-Sud XI University, Laboratoire Aimé Cotton, Bat.505, Orsay, France; 4 Department of Pathology, North Hospital, Aix-Marseille University, Marseille, France; 5 Department of Radiology, Timone Hospital, Aix-Marseille University, Marseille, France; University of Pennsylvania, UNITED STATES

## Abstract

**Background:**

Pre-therapeutic pathological diagnosis is a crucial step of the management of pulmonary nodules suspected of being non small cell lung cancer (NSCLC), especially in the frame of currently implemented lung cancer screening programs in high-risk patients. Based on a human *ex vivo* model, we hypothesized that an embedded device measuring endogenous fluorescence would be able to distinguish pulmonary malignant lesions from the perilesional lung tissue.

**Methods:**

Consecutive patients who underwent surgical resection of pulmonary lesions were included in this prospective and observational study over an 8-month period. Measurements were performed back table on surgical specimens in the operative room, both on suspicious lesions and the perilesional healthy parenchyma. Endogenous fluorescence signal was characterized according to three criteria: maximal intensity (Imax), wavelength, and shape of the signal (missing, stable, instable, photobleaching).

**Results:**

Ninety-six patients with 111 suspicious lesions were included. Final pathological diagnoses were: primary lung cancers (n = 60), lung metastases of extra-thoracic malignancies (n = 27) and non-tumoral lesions (n = 24). Mean Imax was significantly higher in NSCLC targeted lesions when compared to the perilesional lung parenchyma (p<0,0001) or non-tumoral lesions (p<0,0001). Similarly, photobleaching was more frequently found in NSCLC than in perilesional lung (p<0,0001), or in non-tumoral lesions (p<0,001). Respective associated wavelengths were not statistically different between perilesional lung and either primary lung cancers or non-tumoral lesions. Considering lung metastases, both mean Imax and wavelength of the targeted lesions were not different from those of the perilesional lung tissue. In contrast, photobleaching was significantly more frequently observed in the targeted lesions than in the perilesional lung (p≤0,01).

**Conclusion:**

Our results demonstrate that endogenous fluorescence applied to the diagnosis of lung nodules allows distinguishing NSCLC from the surrounding healthy parenchyma and from non-tumoral lesions. Inconclusive results were found for lung metastases due to the heterogeneity of this population.

## Introduction

For early stages non small cell lung cancer (NSCLC) or solitary pulmonary metastasis, surgical resection is usually recommended, though stereotactic radiotherapy or radiofrequency ablation can be seen as valid alternative treatment options [1,2]. In all cases, however, pre-treatment histology is mandatory. Depending on the size and location of the targeted lesion, tissue diagnosis can be achieved through various minimally invasive investigations such as echo-guided transbronchial and CT scan-guided needle biopsies, or thoracoscopic wedge resections. Each procedure, however, encompasses several technical difficulties and limitations. Moreover, those procedures are time-consuming, have an economic impact and more importantly, can lead to substantial morbidity and even mortality [3]. As a result, innovative methods aiming at improving their accuracy still need to be developed. In particular, a precise *in vivo* localization of the targeted lesion remains critical. The clinical relevance of this statement is all the more obvious that the number of small lung opacities is expected to increase dramatically as the result of the worldwide implementation of low-dose CT-scan lung cancer screening programs for high-risk patients [4,5].

Auto-fluorescence is the property of specific endogenous molecules, such as elastin, to send a fluorescent signal after being excited with another luminous signal of lesser wavelength [6]. To the best of our knowledge, such strategy has never been assessed in the setting of lung malignancies.

In this prospective and observational trial, we hypothesized that assessment of endogenous fluorescence in lung tissue with a simple embedded system would be able to distinguish malignant lesions from the surrounding healthy lung parenchyma.

## Methods

The experimental protocol was approved by the ethic committee of the French Society of Thoracic and Cardiovascular Surgery (CERC-SFCTV-2014-1-17-19-9-42-DJXa).

A written informed consent was obtained in all enrolled patients.

### Study design

Over an 8-month period, all consecutive patients referred to our department for surgical management of suspected or known primary or secondary pulmonary malignancies were prospectively included. Exclusion criteria were: lesions less than 5mm wide, previous chemotherapy and carcinoid tumours. Technical problems during the experiment also led to exclusion from the trial.

Measurements were done by the same observer in the operating room, back table, immediately after lung resection, respecting asepsis requirements. Spectroscopic measurements were performed on the surgical specimen before sending it to the Pathology department for examination. Measures were performed on both atypical (wedge) and on anatomical lung resections (lobectomy or pneumonectomy). The information obtained with these measurements did not alter the patients’ management.

On each sample, five spectroscopic measurements were performed on the targeted lesions, five on deflated perilesional lung tissue, and, when possible, 5 more on perilesional ventilated lung tissue, in order to mimic the usual state of the lung during bronchoscopic or CT-scan guided procedures. Nodule location was assessed through surgical specimen palpation. When possible, a tissue sample of the targeted lesion was removed, with a biopsy punch wrapping the needle, at the site of the fifth measurement, for further pathological examination.

### Description of the device

The main goal was to characterize the endogenous fluorescence of pulmonary cancerous lesions, whether primary or secondary. In order to achieve it, the Probea prototype (patent n°W02011010063), previously described by Alchab et al [7], was used. The prototype is composed of two distinct parts. The disposable part is a 25-Gauge needle containing an optical fiber connected to a fixed fluorescence data processing system. A laser diode in the fixed part of the device produces a 405nm-blue light that is brought into contact with cells through the fibered needle. Once excited, endogenous fluorophores contained in the cells emit fluorescence that is gathered by the fibered needle and analyzed with a specific software (ProbeaSoft). This software allowed the recording of 25 fluorescence intensities second with an acquisition time of 4ms for each measurement. The laser light was set at 405nm, which corresponds to the best compromise between an innocuous stimulation of the tissue for patient safety (wavelength above 360 nm) and the optimal one to excite most endogenous fluorophores.

Needles were disinfected with the same protocol between each surgical specimen. Four consecutive one minute-baths were applied: 1) RBS (2%, Carl Roth, Germany); 2) RBS 2%; 3) ethanol; 4) water. Finally, to eliminate all remaining particles potentially compromising the following measurements, the laser remained activated through the needle during fifteen minutes.

Before each series of measurements on surgical samples, Probea was calibrated with the following process: control of the laser beam output power, assessment of the fluorescence of reference colored materials, and record of background signal measured in a dark chamber in order to subtract it from the fluorescence measurements.

### Spectroscopic measurements

**Fig 1:**
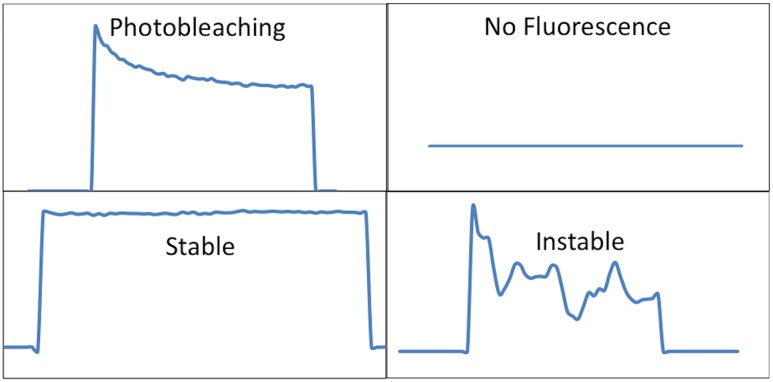
Shape of fluorescent signals.

Analysis of the fluorescent signals were based on three criteria’s:

1. Maximal fluorescence intensity measured (Imax_(λ)_ arbitrary unit, a.u) corresponding to the highest intensity value for each measurement.
2. Emission wavelength (nm) at which this maximal intensity was emitted.
3. Shapes of signals: instable, no fluorescence, stable and photobleaching (Fig 1).

Based on these characteristics we evaluated the signal emitted by lung tissue on:

- The mean maximal fluorescence intensity or Imax (Mean value of the Imax of the five measurements performed on each sample)
- The mean emission wavelength associated with maximal intensity
- The shape of the signals.

Two investigators, including the observer performing the measurements, reviewed all spectra independently. When agreement was not reached concerning the shape of the signal, a third investigator acted as an arbitrator.

### Statistical Analysis

Data were analyzed using the SPSS 17.0 package (SPSS, Chicago, IL). The results were expressed as the mean ± standard deviation (SD) or median (range) for quantitative variables and as percentage for qualitative variables. The Mann-Whitney test was used for non-parametric variables. The Pearson χ2 or Fischer exact test were applied for qualitative variables.

## Results

### Patients

We performed measurements on 129 patients who presented 146 lung lesions. Thirty-three patients were excluded: 8 for documented technical problems with the device, 6 who had carcinoid tumours, 14 who received previous chemotherapy and 5 who had lesions less than 5mm wide.

Fluorescence was measured on 96 patients who presented 111 lesions. Measurements on the targeted nodule were obtained in all patients. Measurements on peripheral lung tissue were unavailable for 2 patients. Measurements on inflated peripheral lung tissue could be performed for 41 patients (205 measurements), 5 of them associated with a benign lesion, 32 with a primary lung cancer and 4 with metastases.

**Fig 2:**
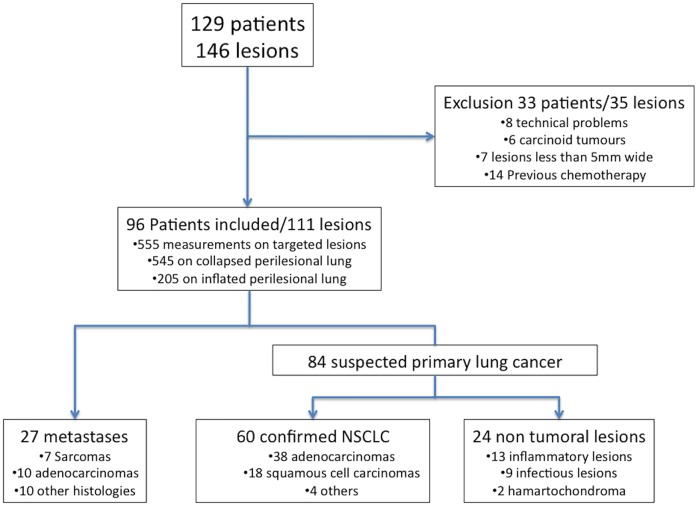
Flowchart.

Pathological reports of the 96 patients are summarized in Fig 2 (and S1 Fig). Eighty-four lesions were pre-operatively suspected of NSCLC. The diagnosis was confirmed for 60 of them (38 adenocarcinoma, 18 squamous cell carcinoma, 3 undifferentiated cancer and one large cell neuroendocrine carcinoma). Concerning the 24 non-malignant lesions, histologies were miscellaneous including infectious and inflammatory lesions or benign tumours. Twenty-seven lesions were diagnosed as metastases on final pathological examination.

#### Suspected or known primary lung cancer

There was no statistical difference on clinical data between patients with a non-malignant lesion when compared to patients presenting with a NSCLC (Table 1).

**Table 1:** Patients’ characteristics.

	Non-tumoral lesions	NSCLC	Statistical analysis^a^	Lung Metastases	Statistical analysis^b^
**Number of patients**	22	57		17	
**Mean Age**	65	63	0,7	64	0,6
**Sexe**					
**Female**	8 (33%)	17 (27%)	0,7	9 (53%)	0,08
**Male**	14 (67%)	40 (73%)	0,8	8 (47%)	0,08
**BMI**	25,7	25,2	0,7	24,5	0,8
**Smokers**	18 (82%)	51 (89%)	0,8	8 (47%)	<0,001
**Non-Smokers**	4 (18%)	6 (11%)	0,5	8 (47%)	<0,001
**Smoking status Unknown**	0	0		1 (6%)	
**Number of Lesions**	24	60		27	
**Number of surgical resections**	23	59		26	
**Non anatomical**	18 (78,3%)	2 (3,4%)	<0,0001	15 (58%)	<0,0001
**Open surgery**	7	1		8	
**Video assisted surgery**	11	1		7	
**Anatomical resection**	5 (21,7%)	48 (81,4%)	<0,0001	11 (42%)	<0,001
**Open surgery**	1	17		4	
**Video assisted surgery**	4	31		7	
**Pneunomectomy or enlarged resection**	0	9 (15,3%)	0,1	0	0,06
**pTNM status**					
**Stage I**	NA	36 (63%)	NA	NA	NA
**IA**		20			
**IB**		16			
**Stage II**	NA	12 (21%)	NA	NA	NA
**IIA**		4			
**IIB**		8			
**Stage IIIA**	NA	8 (14%)	NA	NA	NA
**Unavailable**		1			
**Post-operative treatment**	No	19 (33%)		4 (24%)	
**Mean time of follow-up (days)**	45	71		41	

^a^. Comparison of NSCLC with non-tumoral lesions using the Pearson χ^2^ or the Fischer exact test when necessary. Statistical significance p<0,05.

^b^. Comparison of NSCLC with lung metastases using the Pearson χ^2^ or the Fischer exact test when necessary. Statistical significance p<0,05.

NA: Not applicable. NSCLC: Non Small Cell Cancer.

**Fig 3:**
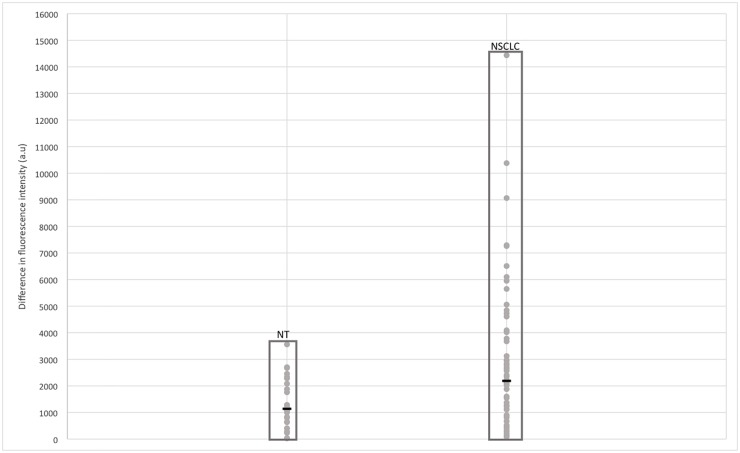
Difference of Mean Imax in absolute value of non-tumoural lesions and non small cell lung cancer with the associated perilesional lung parenchyma. Each dot was calculated with the mean Imax of the five measurements performed on the non-tumoural lesions (n = 24) and NSCLC (n = 60). The black bar corresponds to the median value of each subgroup. NSCLC: Non Small Cell Lung Cancer. NT: Non tumoural.

**Fig 4:**
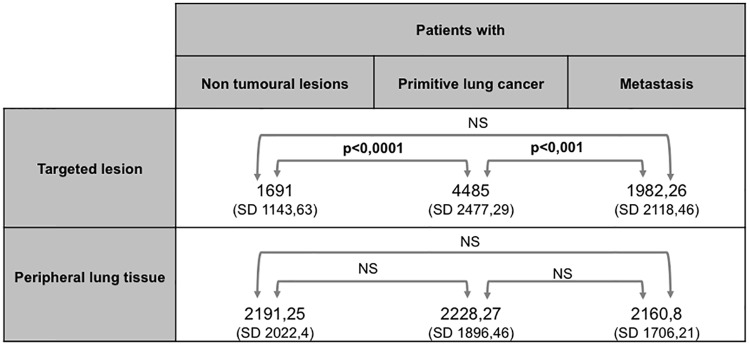
(A) Comparison of mean Imax of the targeted lesions. (B) Comparison of mean Imax of perilesional lung in the different populations. Mean Imax was calculated with the mean Imax of each lesion: 60 NSCLC, n = 24 non-tumoral lesions, 27 metastases. Mean Imax of perilesional lung was calculated separately for each population. Statistical analysis were realised with the Mann-Whitney test, SPSS 17.0 package (SPSS, Chicago, IL). Statistical significance defined as p<0,05. NSCLC: Non Small Cell Lung Cancer. SD: Standard Derivation.

In ultimately proven NSCLC patients, mean Imax was of 4485 ± 2477 (range 45–17755) in the targeted lesion, whereas it was of 2228,27 ±1896 (range 45–10255) in the perilesional lung parenchyma. For the 32 samples for which inflation of perilesional lung was possible, mean Imax was of 1721,7 ± 1455 (range 0–5313). In non malignant lesions, mean Imax was of 1691 ± 1143 (range 211–4835) in the targeted lesion, whereas it was of 2191,25 ±2022,4 (range 0–7538) in the collapsed perilesional lung, and of 890 ± 755 (range 174–1785) in the inflated perilesional lung, when available (Table 2 and Fig 3). Mean Imax of the perilesional lung was not statistically different whether it was associated with non-malignant lesion or a NSCLC (Fig 4).

**Table 2:** Comparison of Imax of perilesional lung and their associated lesions.

	Perilesional lung tissue	Associated lesion	statistical analysis^a^
**Non tumoral lesions n = 24**	^b^2191,25 (± 1706,21)	1691 (± 1143,63)	p = 0,7
**NSCLC n = 60**	^b^2228,27 (± 2022,4) ^c^1721,7 (±1455)	4485 (± 2477,29)	p<0,0001 p< 0,0001
**Lung Metastases n = 27**	^b^2160,8 (± 1896,46)	1982,26 (± 2118,46)	p = 0,3

^a^. Mann-Whitney test, SPSS 17.0 package (SPSS, Chicago, IL). Statistical significance defined as p<0,05.

^b^. Deflated Perilesional tissue.

^c^. Inflated Perilesional tissue, 32 samples available.

NSCLC: Non Small Cell Lung Cancer.

Mean Imax of NSCLC was significantly higher than Imax of the perilesional lung (p<0,0001) (Table 2). Mean Imax of NSCLC was significantly higher than Imax of non-malignant lesions as well (p<0,0001) (Fig 4).

No statistical difference was found between the respective mean Imax values of non-malignant lesions and their perilesional lung parenchyma (Table 2).

**Fig 5:**
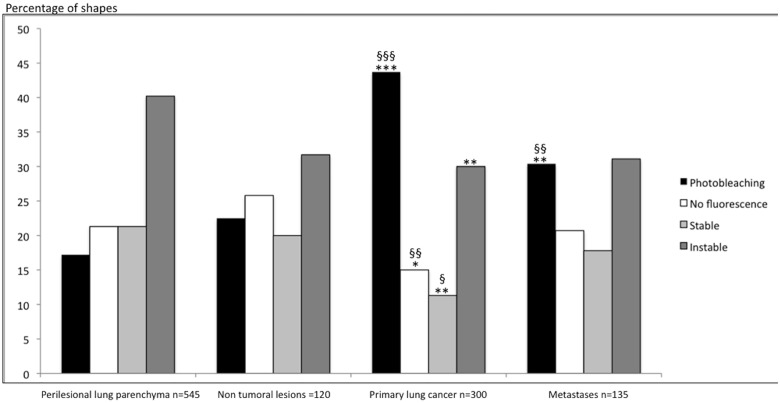
Percentage of shapes of Fluorescent Signals observed in perilesional lung parenchyma, NSCLC, non-tumoral lesions and lung metastases. Comparison of the percentage of each shape between perilesional lung and targeted lesions. Statistical analysis using the Pearson χ^2^ test. Statistical significance defined as * p<0,01, ** p<0,001, ***p<0,0001. Comparison of the percentage of each shape between NSCLC and non-tumoral lesions and lung metastases. Statistical analysis using the Pearson χ^2^. Statistical significance defined as ^§^p = 0,02, ^§§^p<0,01, ^§§§^p<0,001. NSCLC: Non Small Cell Lung Cancer.

Regarding the shape of signals, photobleaching was the main signal in NSCLC. It was significantly more frequent in NSCLC (43,7%), than in perilesional lung tissues (17,2%, p<0,0001) (Table 3). On the other hand, all other types of signals were significantly less frequent in NSCLC than in the perilesional lung (Fig 5). Regarding signal shapes, no difference was found between non-malignant lesions and the perilesional lung (Table 3 and Fig 5).

**Table 3:** Shape of Fluorescent signals in the perilesional lung and the associated targeted lesions. NSCLC: Non Small Cell Lung Cancer. PB: Photobleaching.

	Perilesional lung^a^ n = 545	Non tumoral lesions n = 120	NSCLC^b^ n = 300	Lung Metastases n = 135
**PB**	94 (17,2%)	27 (22,5%)^§§§^	131 (43,7%)***	41 (30,4%)^§§/^**
**No fluorescence**	116 (21,3%)	31 (25,8%)^§§^	45 (15%)*	28 (20,7%)
**Stable**	116 (21,3%)	24 (20%)^§^	34 (11,3%)**	24 (17,8%)
**Instable**	219 (40,2%)	38 (31,7%)	90 (30%)**	42 (41,1%)

^a^. Comparison of the percentage of each shape between perilesional lung parenchyma and targeted lesions. Statistical analysis using the Pearson χ^2^. statistical significance defined as * p<0,01, ** p<0,001, ***p<0,0001.

^b^. Comparison of the percentage of each shape between NSLC and non-tumoral lesions and lung metastases. Statistical analysis using the Pearson χ^2^. Statistical significance defined as ^§^p = 0,02, ^§§^p<0,01, ^§§§^p<0,001.

Photobleaching was statistically more frequent in NSCLC than in non-malignant lesions (p<0,001). Though signals with either a stable shape or no fluorescence were significantly less frequent in NSCLC than in non-malignant lesions (p = 0,02 and p<0,01, respectively), no statistical difference was found for instable signals.

Mean wavelength was of 506 ± 8 nm in NSCLC, (range 498–543), 506 ±10 nm in non-malignant lesions (range 496–535). Overall mean wavelength was of 506 ±10 nm in perilesional lung (range 480–543). No difference was found between wavelength in targeted lesions and corresponding the perilesional lung.

Based on our results, we have drawn the following algorithm including both Imax and shape of signals:

- When mean Imax was higher than 3800, and/or the sum of Imax of signals with photobleaching was higher than 7500, targeted lesions were predicted as NSCLC.
- When targeted lesions did not answer those requirements, the lesions were predicted to be non-tumoral lesions.

Based on this algorithm, among the 24 non-tumoral lesions, 22 were well classified. Among the 60 NSCLC, 46 lesions respected the algorithm (Table 4). The sensitivity and specificity were respectively of 77% and 92%, and the positive predictive and negative predictive values were respectively of 96% and 61%.

**Table 4:** Sensitivity and specificity for primary lung cancer. The sensitivity and specificity were respectively of 77% and 92%. The positive and negative predictive values were respectively of 96 and 61%. NSCLC: Non Small Cell Lung Cancer. PB: Photobleaching.

Definitive Pathology	Imax<3800 and/or PB<7500	Imax≥3800 and/or PB≥7500	
Non-tumoral	22	2	24
NSCLC	14	46	60
	36	48	

Regarding pathological findings, the state of the underlying tissue did not seem to influence the Imax value. The main difference between non-tumoral lesions and primary lung cancer seemed to be fibrosis and necrosis more often found in NSCLC (Table 5 and S1 Fig).

**Table 5:** Comparison of pathological findings between the non-tumoral lesions and NSCLC. NSCLC: Non Small Cell Lung Cancer.

		Non-tumoral lesion n = 24	NSCLC n = 60	Statistical analysis^a^
Targeted lesion	Inflammation	13	35	0,077
	Necrosis	4	31	0,015
	Fibrosis	6	40	p<0,001
Surrounding tissue	Emphysema	13	40	0,186

^a^. Comparison of pathological characteristic between non-tumoral lesion and NSCLC using the Pearson χ^2^ or the Fischer exact test when necessary. Statistical significance p<0,05.

#### Metastases

There were 10 metastases of adenocarcinomas, 7 metastases of sarcomas and 10 metastases of various histologies. Statistical difference was not achieved when comparing sex ratio between patients operated on for lung metastases versus patients operated on for a NSCLC, though there were significantly more smokers in this last group when compared to patients with lung metastases (p<0,001) (Table 1).

**Fig 6:**
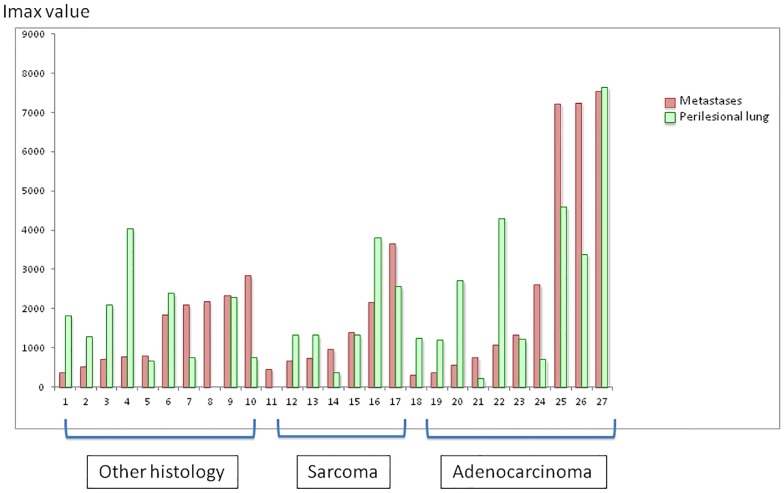
Mean Imax of lung metastases and associated perilesional lung. Mean Imax was calculated using the Imax of the 5 measurements performed on Metastases (n = 27) and perilesional lung parenchyma (n = 25).

Mean Imax in metastases was of 1982,26 ±2118 (range 299–7539) whereas it was of 2160 ± 1706 (range 210–7644) in the perilesional lung (Fig 6). No statistical difference was found between metastases and either the perilesional lung, or non-malignant lesions (Fig 3). Main statistical difference in Imax was found between metastases and NSCLC, with a higher Imax in primary lung cancer (Fig 3).

Regarding signal shapes, photobleaching was statistically more frequent in metastases (30,4%) than in the perilesional lung (17,2%, p<0,001). There was no statistical difference between other shapes of signal (Table 3 and Fig 5). Photobleaching was significantly less frequent in metastases than in NSCLC (p<0,01). No significant difference was found between other shapes of signal, when metastases were compared to NSCLC. Neither photobleaching nor other shapes of signal showed statistical difference between metastases and non-malignant lesions.

The average wavelength was of 504±12 nm (range 498–547) in metastases and was not significantly different from the wavelength in the perilesional lung (506 ± 8 nm; range 497–525).

## Discussion

Auto-fluorescence has been used for several years as a diagnostic tool in lung cancer [8,9]. Tumoral bronchial epithelium is known to present with less fluorescence than normal bronchial epithelium, and those characteristics are used both in staging, follow-up and treatment of bronchial lung cancer [10]. Moreover, though less widely studied and still belonging to experimental protocols, auto-fluorescence of body fluids is an interesting field of research, whether it is applied to lung cancer or other cancer types [11]. Furthermore other studies are ongoing using the fluorescence properties of malignant lesions, but always after injection of an enhancing exogenous product [12–14]. In the literature, numerous side effects are reported when fluorescent tumour-specific contrast agents are used. If side effects of fluorescent contrast agent are admitted during the treatment, they are not acceptable during the pre-therapeutic period when a diagnosis must be obtained, which is the main goal of the Probea device. As an example, during surgery, the use of fluorescent markers such as methylene blue can induce tissue necrosis [15]. In photodynamic therapy, photosensitizing agents such as porphyrins or ALA are used, and can be involved in a major side effect, photosensitivity, lasting 4 to 6 weeks. If patients do not avoid sunlight during this period, they suffer from photosensitivity dermatitis, show local redness, swelling and noticeable scurf [16]. Furthermore, the specificity of these fluorescent agents is reported as low, and do not encourage their use currently [17–19]. Auto-fluorescence of lung tissue, normal or malignant, without adjunction of any product, has not been described outside of the bronchial tree. Endogenous fluorescence has its own limits, but also has the enormous advantage of no toxicity for the patient, which fully justifies its use in a diagnostic approach, as a supplementary tool of those currently used.

We chose not to study NSCLC with pre-operative chemotherapy because of the potential modifications of structure in malignant lesions subsequent to this treatment. Moreover, those lesions did not match the goal of our study from a clinical point of view. For similar reasons we did not include carcinoid tumours. Those tumours are uncommon, and usually have an indolent evolution. The design of the study did not plan the inclusion of enough carcinoid tumours to characterize specifically their fluorescence signal.

Endogenous fluorophores, naturally present in tissues, could be the mirror of biological modifications caused by cancer development. Hanahan and Weinberg described that cancer causes changes in both structural proteins of the extracellular matrix and cellular metabolism [20]. These phenomena may induce changes in NADPH and FAD expression, involved in cellular metabolism, and in concentrations of structural proteins such as collagen and elastin, leading to modified auto-fluorescence signals. The lung is an elastic organ composed of 28% of elastin fibers that presents a maximal excitation wavelength of 375nm (300–410 nm) and a maximal emission wavelength of 520nm [21]. Richard and Muraka identified that bonds created between desmosin and isodesmosin in tropoelastin fibers are responsible for elastin fluorescence. The authors have shown that modifications on elastin concentration and structure could be good markers to evaluate biological changes [22]. We hypothesized that modifications of elastin fibers, in either structure or concentration, were responsible for the difference of fluorescence seen between perilesional lung, non-malignant lesions, metastases and NSCLC. Furthermore, other studies have demonstrated that fluorescence was different between necrotic and healthy tissue [23]. This is coherent with our results, where among our samples of NSCLC, the most common histological finding associated with lack of fluorescence was necrosis (S1 Fig).

We found that spontaneous fluorescence of NSCLC, regardless of the histological subtype, was higher than either perilesional lung or non-malignant lesions. Those results contrast with what was reported in auto-fluorescence bronchoscopy of malignant lesions [8,9]. Lack of fluorescence compared to normal bronchial epithelium is, in part, a consequence of the absorption of excitation and fluorescence light by the cancerous lesion, secondary to the more important thickness of its epithelium [10]. Contrary to what was done in bronchoscopy studies, we did not use probes to measure the fluorescence, but needles directly inserted in the tumoral stroma. Thus the targets of our measurements were different, which could explain the difference in our results.

For NSCLC, our results suggest that endogenous fluorescence differs according to the histological sub-type. For squamous cell carcinomas, signals presented a higher Imax than adenocarcinomas, but the shape of signals seemed to be more instable. We hypothesized that the differences observed in the signal shapes were secondary to the more frequent presence, and in a more abundant way, of necrosis in squamous cell carcinomas.

Regarding subclasses of adenocarcinoma, we had 7 ground-glass lesions on pre-operative CT-scan, with a total or partial aspect of ground-glass opacity. Five of these lesions were predicted to be primary lung cancer based on our algorithm and were finally confirmed as true adenocarcinoma with a lepidic contingent on pathological reports. The other two samples predicted as non-tumoral were confirmed as true non malignant lesions on the final pathological report. Because the number of ground-glass opacities are expected to increase with low dose CT-scan lung cancer screening program, these findings represent a promising result for further investigations, taking into account that 90% of these lesions remain benign.

Though not available for all samples, Imax in the inflated perilesional lung was lower than in the collapsed perilesional lung. We speculate that when tested in conditions where the lung is ventilated, for example during percutaneous biopsies, the difference in fluorescent signals between primary lung tumours and the perilesional lung would increase.

We did not find any difference between signals in lung metastases and the perilesional lung, except in the shapes of signals where photobleaching was more frequent in metastases, though statistical significance was not achieved. Our population consisted of a huge range of several types of metastases, and this heterogeneity could explain why we were unable to show any difference in Imax with the perilesional lung parenchyma. When taken separately, metastases of sarcoma and adenocarcinoma (excluding metastases of colorectal cancer) seemed to have fluorescence signals similar to those found in primary lung cancer, associating photobleaching and high Imax. In contrast, metastases of colorectal cancer seemed to have a specific signal, associating low Imax and photobleaching (data not shown). Unfortunately, our sample size was not sufficient enough to allow statistical analysis for each subtype of metastases.

Our current results deserve several points of discussion. First, without injection of any product, we were able to describe a fluorescence signal specific of NSCLC. After stimulation with the laser beam, the returning signal was in favour of a primary lung cancer when a high Imax and photobleaching were associated. On the contrary, when a low Imax and another shape of signal were associated a non-malignant lesion was suspected. Basically, these results constitute a benchmark for other *in vivo* investigations in lung cancer detection. In this context using the Probea device, which distinguishes NSCLC both from peripheral tissue and non-malignant lesions, accuracy of percutaneous and bronchoscopic biopsies could be improved, resulting in less useless surgical procedures. Furthermore, this device could be an asset in therapeutic management as well, when percutaneous radiofrequency pulmonary ablation is chosen, to control the residual tumoral margin at the end of the procedure. The Probea device needs further testing in *in-vivo* conditions, to evaluate its clinical relevance, and its benefit in association with the current diagnostic procedures in lung cancer.

Secondly, in the current context of increasing isolated pulmonary nodules found at low-dose CT-scan screening in patients with a high risk of lung cancer, improvement of diagnostic tools is required. Earlier detection of lung cancer allows better outcome while decreasing cancer-related death [24]. But it also leads to an increase in potentially harmful pulmonary investigations, such as CT-scan-guided biopsies, bronchoscopic biopsies and even surgery. For example, during the study undergone by the US National Lung Screening Trial Research Team, up to 25% of futile surgical procedures were performed [5]. This is particularly true for ground-glass lesions. Currently, only clinical and imaging criteria (CT-scan and PET-scan) are used to estimate the probability of a lung cancer [25]. Harders et al compared results of CT-scan and PET-FDG in the characterization of pulmonary lesions [26]. They found that the accuracy of both modalities were similar with a sensitivity and specificity of 97% and 47% for the PET-scan, and a positive predictive value and negative predictive value of 89% and 79%. In their study PET-FDG had a false-positive rate of 53%. Thus, a high number of these pulmonary nodules, after clinical and imaging evaluation, need further investigations including biopsies [3, 25]. In the present study, the device Probea has demonstrated a high predictive positive value of 96%, and mild negative predictive value of 61%. The negative predictive value of Probea is less that the one found with PET-scan, though it was not the goal to oppose the two techniques but merely to determine if Probea, after PET evaluation, may be an asset to confirm the diagnostic of cancer. In the setting of cancer detection rather than a screening context, the high positive predictive value of the Probea device seems to be of interest to help the clinician to obtain a pathological diagnosis mostly in association with others existing imaging exams.

The invasive procedures currently used to make the proof of a NSCLC are both time-consuming and expensive and carry a substantial risk of adverse events [3,5, 25]. Our current data have to be confirmed on an *in vivo* model before its acceptance in what constitutes the current tools for the diagnostic of lung cancer.

In conclusion, our results provide the first data available on the endogenous fluorescence of pulmonary tissue in a human *ex vivo* model. Our results suggest that endogenous fluorescence applied to the diagnosis of lung nodules, allows differentiating NSCLC from the surrounding healthy lung and from non-malignant lesions. Interesting results were found for metastases, but the heterogeneity of this population requires further investigations. Potential clinical utilisations of this embedded detection are numerous in the fields of surgery, endoscopy and radiology in association with the current invasive diagnostic techniques.

## Supporting Information

S1 Fig Definitive pathological characteristics in the targeted lesions and their associated perilesional lung. NSCLC: Non Small Cell Lung Cancer. SCC: Squamous Cell Carcinoma.
(DOCX)
