# Supplementary material for: Pulmonary Endogenous Fluorescence Allows the Distinction of Primary Lung Cancer from the Perilesional Lung Parenchyma
Source: PLoS One. 2015 Aug 5;10(8):e0134559. doi: 10.1371/journal.pone.0134559 (PMC4526534; doi:10.1371/journal.pone.0134559)
Supplement: S1 Fig — NSCLC: Non Small Cell Lung Cancer. SCC: Squamous Cell Carcinoma. (DOCX) [file pone.0134559.s001.docx]

|  | Definitive Pathology | In the lesion | | | Surrounding tissue | | Predictive algorithm |
| --- | --- | --- | --- | --- | --- | --- | --- |
|  |  | Inflammation | Necrosis | Fibrosis | Emphysema | Other lesions |  |
| 1 | Granuloma compatible with tuberculosis | Yes | Caseous | No | Yes | Several other nodules | NT |
| 2 | Parenchymal node | No | No | No | Yes | Sinusal histiocytosis anthracosis | NT |
| 3 | Aspergilloma | No | No | No | No | Fibro-necrotic lesions | NT |
| 4 | Granuloma | Yes | No | No | No | No lesion | NT |
| 5 | Inflammatory bronchiolitis | Yes | No | Alveolar fibrosis | No | Moderate inflammatory infiltrate | NT |
| 6 | No lesion | No | No | No | No | No lesion | NT |
| 7 | 19 mm granuloma compatible with tuberculosis | Yes | Yes | No | No | Pleiomorphic inflammatory infiltrate | NT |
| 8 | 12 mm Anthracosis nodule | Lymphoid stroma | Yes | No | No | No lesion | NT |
| 9 | 3 cm hamartochondroma | No | No | No | No | No lesion | NT |
| 10 | 3 cm interstitial pneumopathy | Yes | No | No | Focal emphysema | Collagenic fibrosis | NT |
| 11 | 6 mm hamartochondroma | No | No | Yes | Yes | No lesion | NT |
| 12 | Acute pneumopathy | No | No | No | Yes | Siderophagic alveolitis | NT |
| 13 | 13mm granuloma | Yes | No | No | Yes | No fibrosis | NT |
| 14 | 7 mm granuloma compatible with tuberculosis | Yes | Caseous | Fibrotic capsule | Yes | Several associated lesions | NT |
| 15 | Inflammatory and fibrosis lesions | Yes | No | No | Emphysema with bulla | Fibrosis | NT |
| 16 | No lesion | No | No | No | No | No lesion | NT |
| 17 | Aspecific inflammatory lesions | Yes | No | Yes | No | Macrophagic inflammation | NT |
| 18 | Granuloma | Yes | No | No | Histological emphysema | No lesion | NT |
| 19 | Aspergilloma | Yes | No | Collagenic fibrosis | No | Lymphatic elements | NT |
| 20 | No lesion | No | No | No | Yes | Macrophagic inflammation | NT |
| 21 | 12mm infarcted lesion | No | No | No | Yes | Anthracosis remodelling | NT |
| 22 | Diffuse sero-hematic lesions | No | No | No | Yes | Hemorrhagic lesions | NT |
| 23 | 15mm lymphatic node | No | No | No | No | No lesion | NSCLC |
| 24 | 6 mm lesion with fibro-inflammatory remodelling | Yes | No | Collagenic and elastosis fibrosis | Histological emphysema | No lesion | NSCLC |
| 25 | 80 mm badly differentiated SCC | No | Necrotic remodelling | Fibro-collagenic stroma | Histological emphysema with bulla | No lesion | NT |
| 26 | 50 mm SCC with mild differentiation | Yes | Focal necrosis | No | No | No lesion | NT |
| 27 | 31 mm well differentiated endobronchial SCC | Yes | Yes | Fibrotic stroma | Yes | No Lesion | NT |
| 28 | 23 mm badly differentiated adenocarcinoma | No | Important necrotic remodelling | No | Yes | Second lesion | NT |
| 29 | 10 mm adenocarcinoma | No | No | No | No | Absence of differentiation | NT |
| 30 | 21 mm solid adenocarcinoma | Discreet | Focal necrosis | No | Yes | Anthrocosic remodelling | NT |
| 31 | 25 mm SCC with mild differentiation | Mild | Yes | No | Emphysema with bulla | Mucoid Impacts anthracosis | NT |
| 32 | 14mm adenocarcinoma sarcomatoid contingent | Yes | No | Fibrotic stroma | No | Anthracosis and hemorrhagic remodelling | NT |
| 33 | 50 mm badly differentiated SCC | Yes | Necrotic remodelling | Fibrotic stroma | No | No lesion | NT |
| 34 | 22 mm solid adenocarcinoma | No | No | No | Yes | Atypical cells | NT |
| 35 | 7 mm well differentiated acinar adenocarcinoma | No | No | Fibro-elastosis stroma | Yes | No lesion | NT |
| 36 | 120 mm tumour without differentiation | No | Yes | No | No | No lesion | NSCLC |
| 37 | 15 mm solid badly differentiated adenocarcinoma | Mild | No | Fibro-elastosic stroma | Yes | No lesion | NT |
| 38 | 38 mm large cell neuroendocrine carcinoma | No | Necrotic remodelling | Fibrosis | Emphysema with bulla | No lesion | NSCLC |
| 39 | 12 mm acinar and papillary adenocarcinoma | Yes | No | Fibrotic stroma | Yes | Hemorrhagic remodelling and scar fibrotic remodelling | NT |
| 40 | 15 mm well differentiated acinar adenocarcinoma | No | No | Yes | Emphysema with bulla | No lesion | NSCLC |
| 41 | 25 mm badly differentiated adenocarcinoma | No | Important necrotic remodelling | No | Emphysema with bulla | No lesion | NT |
| 42 | 15 mm adenocarcinoma | Yes | No | Fibrotic stroma | Yes | No lesion | NSCLC |
| 43 | 35 mm acinar adenocarcinoma | Yes | No | No | No | No lesion | NSCLC |
| 44 | 35 mm badly differentiated SCC | Yes | Necrotic remodelling | Fibrotic stroma | Histological emphysema | Anthracosic remodelling | NSCLC |
| 45 | 14 mm, well differentiated SCC with keratine | Yes | Necrotic remodelling | Fibrotic stroma | Yes | Anthracosis remodelling | NSCLC |
| 46 | 12 mm adenocarcinoma with a 5 mm in situ contingent | No | No | Fibrotic stroma | Yes | Second lesion | NSCLC |
| 47 | 42 mm badly differentiated SCC | No | Yes | Fibro-collagenic stroma | Yes | No lesion | NSCLC |
| 48 | 45 mm acinar adenocarcinoma | Yes | Yes | Fibrotic stroma | No | Collagenic Fibrosis | NSCLC |
| 49 | 6 mm acinar adenocarcinoma | No | No | No | Yes | No lesion | NSCLC |
| 50 | 19 mm solid and acinar adenocarcinoma, badly differentiated | No | Focal necrosis | No | No | No lesion | NSCLC |
| 51 | 30 mm badly differentiated carcinoma | Yes | No | Fibrotic stroma | Yes | No lesion | NSCLC |
| 52 | 15 mm adenocarcinoma | No | No | Fibrotic stroma | Yes | 2 peripheral nodules | NSCLC |
| 53 | 14mm acinar and badly differentiated adenocarcinoma | Acute intra-tumoral inflammation | No | Fibrotic stroma | Focal emphysema | Vascular neoplastic emboli | NSCLC |
| 54 | Badly differentiated adenocarcinoma | Yes | Yes | Fibroblastic fibrosis | Yes | Anthracosic remodelling | NSCLC |
| 55 | 23 mm badly differentiated SCC | Yes | Necrotic remodelling | Fibrotic stroma | Focal Emphysema | No lesion | NSCLC |
| 56 | 9 mm well differentiated adenocarcinoma with a lepidic contingent | Yes | No | Fibrotic stroma | Yes | No lesions | NSCLC |
| 57 | 100 mm mucinous adenocarcinoma with lepidic contingency | No | Yes | No | No | Anthracosis | NSCLC |
| 58 | 20 mm acinar adenocarcinoma | Yes | Yes | No | No | No lesion | NSCLC |
| 59 | 45 mm acinar and papillary adenocarcinoma | Yes | No | Fibrotic stroma | No | Scar tissue Fibro-elastosis remodelling | NSCLC |
| 60 | 39 mm well differentiated SCC | Yes | Necrotic remodelling | Collagenic fibrosis | Yes | Anthracosis remodelling | NSCLC |
| 61 | 10 mm badly differentiated acinar adenocarcinoma | Yes | Focal necrosis | Fibrotic stroma | Yes | No lesion | NSCLC |
| 62 | 40 mm papillar adenocarcinoma with peripheral lepidic contingency | No | No | No | Yes | Elastosis stroma | NSCLC |
| 63 | Solid 7 mm badly differentiated adenocarcinoma | Mild | No | Fibro-elastosic stroma | Yes | No lesion | NSCLC |
| 64 | 12 mm SCC | Yes | No | Fibro-elastosic center | Yes | Anthracosis | NSCLC |
| 65 | Well differentiated 27 mm adenocarcinoma | Yes | Yes | Fibro-inflammatory remodelling | No | No lesion | NSCLC |
| 66 | 9 mm acinar and papillary adenocarcinoma | No | No | No | No | No lesion | NSCLC |
| 67 | 35 mm acinar and mucinous adenocarcinoma lepidic contingent | No | No | No | Yes | Elastosic stroma, hemorrhagic remodelling | NSCLC |
| 68 | 30 mm well differentiated SCC with keratine | No | Yes | Fibro-elastosic stroma | Yes | No lesion | NSCLC |
| 69 | 20 mm well differentiated SCC | Yes | Few necrotic remodelling | Fibrotic stroma | Yes | No lesion | NSCLC |
| 70 | 65 mm SCC with mild differentiation | Yes | Yes | Fibrotic stroma | Yes | Hemorrhagic and anthracosis remodelling | NSCLC |
| 71 | 19 mm acinar and papillary adenocarcinoma | No | No | Fibrotic stroma | No | Hemorrhagic remodelling | NSCLC |
| 72 | 10 mm acinar adenocarcinoma | Mild | No | Fibro-elastosic stroma | Yes | Macrophagic inflammation | NSCLC |
| 73 | 6 mm acinar adenocarcinoma | Mild | No | Fibro-elastosic stroma | Yes | No lesion | NSCLC |
| 74 | 40 mm acinar adenocarcinoma | Mild | Yes | Fibro-elastosic stroma | Yes | No lesion | NSCLC |
| 75 | 8 mm acinar adenocarcinoma included in a 25 mm lepidic contingency | No | No | No | No | No lesion | NSCLC |
| 76 | Muco-epidermoid cancer | Yes | Large necrosis | Fibrotic stroma | Yes | Macrophagic alveolitis | NSCLC |
| 77 | 10 mm SCC with mild differentiation | Yes | No | Fibrotic stroma | No | No lesion | NSCLC |
| 78 | 20 mm SCC with mild differentiation | Yes | No | Fibrotic stroma | No | No lesion | NSCLC |
| 79 | 10 mm acinar adenocarcinoma with lepidic contingent | Mild | No | Fibrotic stroma | No | No lesion | NSCLC |
| 80 | 15 mm acinar and papillary adenocarcinoma | No | No | No | No | No lesion | NSCLC |
| 81 | 15 mm acinar and papillary adenocarcinoma | Yes | No | Fibrotic stroma | Yes | Neoplastic emboli | NSCLC |
| 82 | 15 mm well differentiated SCC | No | No | No | No | No lesion | NSCLC |
| 83 | 32 mm badly differentiated adenocarcinoma | No | Necrotic remodelling | Fibrotic stroma | Yes | Hemorrhagic, anthrocosis, elastosis remodelling | NSCLC |
| 84 | 15 mm adenocarcinoma | Yes | No | Fibrosis | Yes | macrophagic alveolitis | NSCLC |
